# The Multifaceted Role of the IL-2 Cytokine Family in Melanoma: Mechanisms, Therapeutic Implications, and Immune Modulation

**DOI:** 10.1155/jimr/8890939

**Published:** 2025-07-02

**Authors:** Mona Daghaighei, Samaneh Dodge, Soheil Bolandi, Boutros Youssef, Niket Attarde, Moein Maddahi, Maryam Mostofi, Reza Morovatshoar, Mehrnaz Mostafavi, Dejbakht Majid, Mohammadamin Joulani, Pegah Tamimi, Malihe Sharafi, Qumars Behfar, Yasaman Ghodsi Boushehri, Alireza Azani

**Affiliations:** ^1^Islamic Azad University of Tehran, Medical Branch, Tehran, Iran; ^2^School of Pharmacy, Shahid Beheshti University of Medical Science, Tehran, Iran; ^3^School of Medicine, Shiraz University of Medical Sciences, Shiraz, Iran; ^4^Department of Cardiovascular Diseases, Faculty of Medical Sciences, Lebanese University, Hadath, Lebanon; ^5^Department of Medicine, Dr D Y Patil Medical College, Navi Mumbai, India; ^6^Department of Medicine, Yeditepe University of Medical Sciences, Istanbul, Türkiye; ^7^Faculty of Medicine, Ondokuz Mayis University, Samsun, Türkiye; ^8^Molecular Medicine Research Center, Hormozgan Health Institute, Hormozgan University of Medical Sciences, Bandar Abbas, Iran; ^9^Faculty of Allied Medicine, Shahid Beheshti University of Medical Sciences, Tehran, Iran; ^10^Gerash Amir-al-Momenin Medical and Educational Center, Gerash University of Medical Sciences, Gerash, Iran; ^11^Student Research Committee, Faculty of Medicine, Iran University of Medical Sciences, Tehran, Iran; ^12^School of Medicine, Tehran University of Medical Sciences, Tehran, Iran; ^13^Department of Biology, Faculty of Basic Sciences, Azarbaijan Shahid Madani University, Tabriz, Iran; ^14^Faculty of Medicine, Tehran University of Medical Sciences, Tehran, Iran; ^15^Department of Medical Genetic, Faculty of Medicine, Tehran University of Medical Sciences, Tehran, Iran

**Keywords:** cytokines, immunotherapy, interleukin, interleukin-2 family, melanoma

## Abstract

**Background and Objective:** Melanoma is a complex malignancy where the interplay between immune cells, cytokines, and the tumor microenvironment (TME) significantly influences disease progression and patient outcomes. This review explores the involvement of the interleukin-2 (IL-2) cytokine family in both the development and therapeutic approaches for melanoma.

**Methods:** A narrative literature review was conducted, synthesizing findings from studies on immune cell behavior, cytokine functions, and their implications in melanoma and other cancers. This narrative review emphasizes the roles of immune cells and cytokines in both promoting and inhibiting tumor growth.

**Results:** Neutrophils, influenced by tumor-derived cytokines, can adopt phenotypes that either inhibit or promote tumor growth. B cells in the TME often correlate with better survival, although their regulatory forms can suppress immune responses. Tissue-resident memory T cells (TRM cells) are crucial for antitumor immunity, particularly in response to immune checkpoint inhibitors (ICIs). Dendritic cells (DCs) are vital for antigen presentation, yet their function can be compromised in melanoma. Macrophages frequently support tumor growth through immunosuppressive actions. The IL-2 cytokine family, including IL-2, IL-4, IL-7, IL-9, IL-15, and IL-21, plays diverse roles in immune regulation. These cytokines are involved in T-cell proliferation, B-cell differentiation, and modulation of other immune responses, influencing both tumor progression and the effectiveness of immunotherapies.

**Conclusions:** Immune cells and cytokines are pivotal in the pathogenesis, progression, and immunotherapy of melanoma. Understanding their complex roles offers insights into potential therapeutic strategies, highlighting the importance of targeted immunotherapies in treating melanoma and possibly other cancers. Additional studies are required to clarify the precise mechanisms and interactions occurring within the TME to enhance treatment strategies.

## 1. Introduction

Skin cancer (SC) is among the most devastating cancers of the past decade and ranks as the fifth most common type of cancer [1]. Predictions indicate that it could become the leading cause of mortality, surpassing heart disease and posing the greatest challenge to increasing life expectancy in the coming years. The International Agency for Research on Cancer's annual status report revealed that in 2018, there were around 9.6 million deaths related to cancer and 18.1 million new cancer cases worldwide [2, 3]. Melanoma, an aggressive and lethal SC originating from melanocytes, poses a serious health risk worldwide [2]. Malignant melanoma alone accounted for 75% of all mortality cases among SC patients [4]. Its development is driven by a complex combination of genetic mutations, environmental influences, and immune system interactions [5]. Despite considerable progress in understanding the molecular mechanisms in the pathogenesis of melanoma, its incidence is steadily increasing globally, highlighting a persistent public health issue [6]. Among the numerous factors that shape the immune response to melanoma, cytokines are of particular interest [7].

The interleukin-2 (IL-2) cytokine family, including IL-2, IL-4, IL-7, IL-9, IL-15, and IL-21, plays a vital role in modulating immune responses and has been extensively studied across a range of cancers [8]. These cytokines are critical for lymphocyte growth, differentiation, and survival, making them essential components of both the innate and adaptive immune systems [9]. IL-2, the first member identified in this family, has been thoroughly studied for its role in cancer [10]. Its main function is to stimulate the proliferation and activation of T cells, which are critical for generating an effective immune response against tumors [11]. IL-2 has demonstrated potential in treating various cancers [12]. High-dose IL-2 therapy has received approval for use in metastatic renal cell carcinoma and metastatic melanoma [13], showing its ability to induce lasting remission in some patients. However, its use is constrained by significant toxicities, highlighting the need for a better understanding of its mechanisms and the development of safer, more effective treatment strategies [14].

In melanoma, IL-2 plays a role in shaping the antitumor immune response and modulating the tumor microenvironment (TME), which may contribute to immune suppression [15]. Recent studies are starting to reveal how IL-2 and its family members impact melanoma progression and treatment response. These findings are providing critical insights into new potential therapeutic targets and strategies [16].

This study aims to conduct an in-depth exploration of how the IL-2 cytokine family influences melanoma. It focuses on reviewing the molecular and cellular mechanisms through which these cytokines impact melanoma development and progression.

## 2. Immunologic Background of Melanoma

### 2.1. Role of Immune Cells in the Pathogenesis of Melanoma

Neutrophils, the most prevalent immune cells in the TME, usually serve as the first line of defense in the innate immune system [17]. However, cancer-derived cytokines such as TGF-β, IL-6, and IL-8 can attract and alter neutrophils, transforming them from an antitumor (N1) to a tumor-promoting (N2) phenotype. N1 neutrophils inhibit proliferation and release high levels of TNF-α, whereas N2 neutrophils, which have a longer lifespan, support cancer growth by producing pro-tumor factors like ROS, neutrophil extracellular traps (NETs), IL-6, IL-8, and MMP-9 [18, 19]. In melanoma patients, a poor prognosis is often associated with a high number of neutrophils. This indicates that tumors can specifically attract these immune cells to promote their own growth and progression [20]. In metastatic melanoma, neutrophils responded to conditioned media (CM) from melanoma cell lines (SKMEL28 and A375) with increased chemotaxis, survival, activation, and NET release, unlike CM from primary melanocytes. Advanced melanoma patients showed higher serum levels of neutrophil-related mediators and NETs compared to healthy controls, suggesting that melanoma cells activate neutrophils and potentially influence tumor progression [21]. Mass cytometry in melanoma patients revealed seven neutrophil clusters, with some subsets decreasing and others increasing during tumor progression. Likewise, in lung cancer, mass cytometry revealed distinct subgroups within LDN and HDN neutrophil populations, with LDN subtypes being particularly abundant in patients with advanced-stage lung cancer. A specific subgroup, marked by CD66b+/CD10 low/CXCR4+/PDL1 intermediate expression, was exclusively detected in advanced lung cancer cases and linked to a worse prognosis [22]. Tumor cells enhance the production of extracellular vehicles (EVs) to interact with the stroma, creating a supportive environment for the tumor. Specifically, EVs originating from human melanoma cells promote the attraction of neutrophils and induce the formation of NETs via the CXCR2/PI3K-AKT signaling pathway [23]. Neutrophil-derived Annexin A1 (AnxA1) contributes to melanoma metastasis. Elevated levels of AnxA1 were detected in the biopsies and serum of melanoma patients, alongside increased neutrophil-lymphocyte ratios. In mice with lung melanoma metastasis, neutrophils exhibited higher AnxA1 levels, which facilitated melanoma cell invasion through FPR pathways. Inhibition of AnxA1 led to a reduction in both metastasis and AnxA1 levels, suggesting its potential as a biomarker for the progression of melanoma [24].

The presence of B cells (CD19+) or the chemokine CXCL13 in tumor tissue has been correlated with improved overall survival (OS) in melanoma patients. Moreover, when B cells are found alongside CD8+ T cells within tumors, survival rates are further increased [25]. B cells are pivotal in melanoma pathogenesis by contributing to the antitumor immune response. In melanoma patients, there is a higher prevalence of memory B cells in tumors compared to blood, and these cells exhibit unique antibody repertoires. These undergo clonal expansion and generate antibodies that show signs of affinity maturation and polyreactivity, suggesting an active autoimmune-like response within the TME. Tumor-derived antibodies can recognize autoantigens, and patients with active melanoma have elevated serum antibody levels. This indicates that B-cell dysregulation and their autoimmune-like characteristics influence the humoral immune response in melanoma [26]. B cells crucially contribute in melanoma pathogenesis by infiltrating tumors and shaping the immune response. Chemokines like CXCL13 in the TME influence B-cell infiltration and the formation of tertiary lymphoid structures (TLSs), which correlate with better survival rates in melanoma patients. However, CXCR5 expression, activated by CXCL13, can promote tumor growth via pathways such as MAPK, PI3K, and RAC1. Additionally, some B cells in the tumor may develop regulatory properties (Bregs) under the influence of the TME, contributing to immunosuppression through cytokines like IL-10, IL-35, and TGF-β [27].

Tissue-resident memory T cells (TRM cells) play a vital role in antitumor immunity in melanoma. They preserve immune balance and defend against tumor cells and primary melanoma development. In the context of metastatic melanoma, TRM cells are prime targets for immune checkpoint inhibitors (ICIs) because they express high levels of PD-1, CTLA-4, and LAG-3. Treatment with ICIs reactivates and expands these cells, resulting in tumor destruction through the secretion of effector molecules such as IFN-γ [28]. Analyzing T-cell responses in melanoma presents significant challenges. By examining T-cell receptor (TCR) data from melanoma patients and healthy controls, it was found that melanoma-associated antigen (MAA)-specific TCRs share sequence similarities that enable the prediction of anti-MAA T cells. These T cells can distinguish melanoma patients from healthy individuals and predict metastatic recurrence. Additionally, anti-MAA T cells interact with regulatory T cells and tumor cells. Treatment with anti-PD1 (+anti-CTLA4) therapy increases the number of expanded anti-MAA clones and reverses their exhaustion phenotype, providing insights into antigen-specific responses [29]. A study on melanoma patients revealed that CD4+ T cells often recognize mutant neoantigens [30]. CD4+ T cells are crucial in melanoma for their direct contribution to tumor control. In a mouse model of cutaneous melanoma, these cells infiltrated tumors, assumed various effector states, and offered protection independently of other lymphocytes. Their antitumor function utilized multiplex cytotoxic pathways, such as TNF and Fas ligand, and triggered IFN-γ–dependent nitric oxide production in myeloid cells. These results underscore the diverse roles of CD4+ T cells in establishing protective antitumor immunity [31].

Throughout melanoma progression, dendritic cells (DCs) are essential in the immunoediting process, which encompasses stages of immune elimination, balance, and evasion [32]. Melanoma cells indirectly suppress their own proliferation through DCs, which are also essential for processes like DC maturation, migration, and cross-priming. However, their ability to interact effectively with cytotoxic T cells through immune checkpoint receptor ligands is diminished. The TME is characterized by the presence of highly proliferative melanoma cells and immune cells such as T-cells, natural killer (NK) cells, tumor-associated macrophages (TAMs), T-regulatory cells (T-regs), myeloid-derived suppressor cells (MDSCs), and endothelial cells, fosters immunosuppression. This environment is rich in tolerogenic factors and cytokines like IL-6 and IL-10, which further inhibit the function of DCs [33, 34].

**Figure 1:**
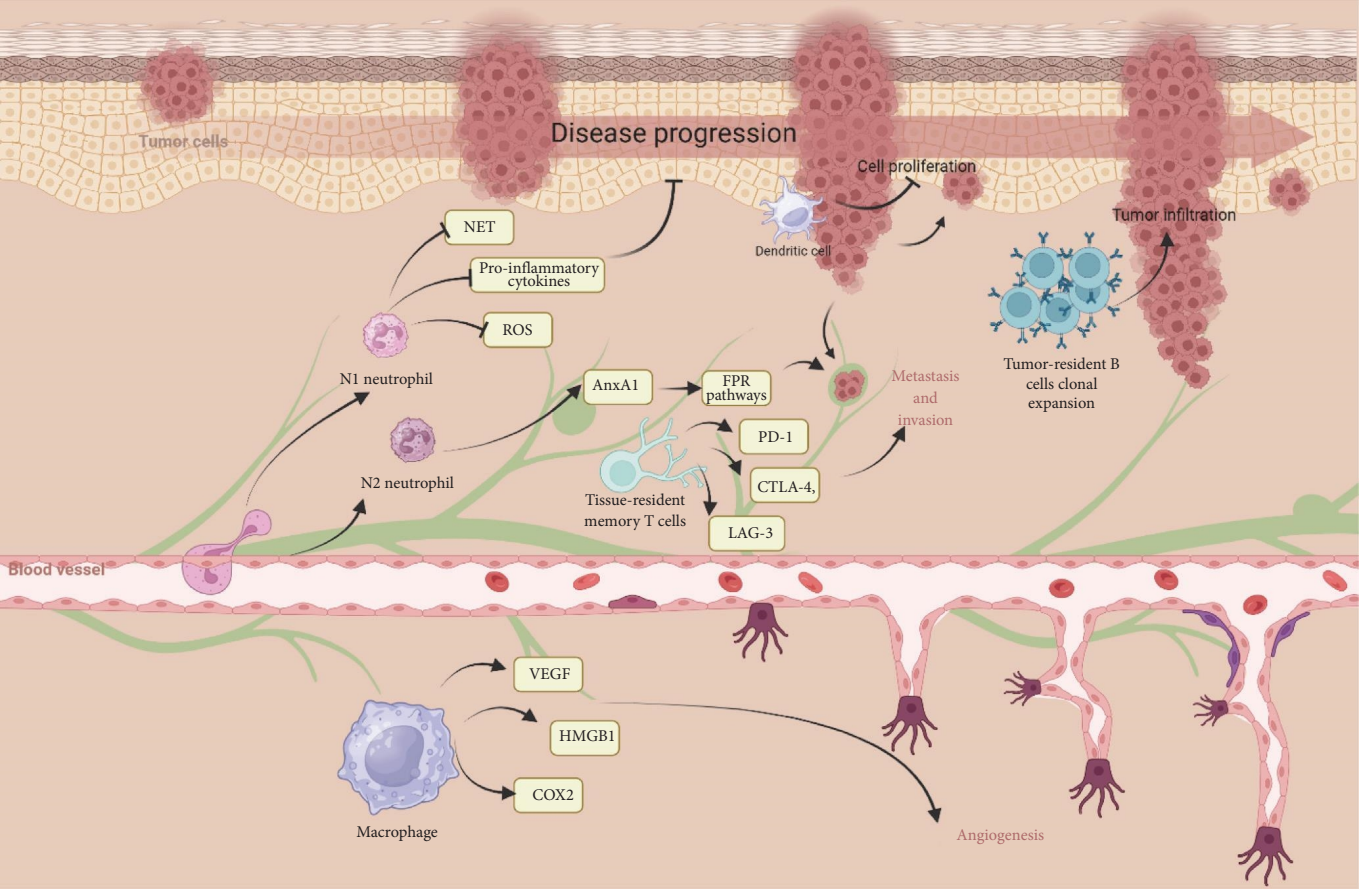
The role of different immune cells in the pathogenesis of melanoma. Created with Biorender.com.

Macrophages significantly contribute to melanoma pathogenesis. Found in both primary and metastatic lesions, they are linked to poor prognosis [35]. These cells interact with melanoma cells through various factors such as GM-CSF and CCL2, CCL8, and CCL15, affecting their recruitment and functionality [36]. Macrophages facilitate tumor growth, invasion, and immunosuppression via pathways involving TNF, Fas ligand, and IL-10 [37]. They also support angiogenesis and create an immunosuppressive TME by producing VEGF, COX-2, and HMGB1 [37]. The STAT3 and TNFR–NF-κB signaling pathways further influence these processes, enhancing M1 and M2 macrophage production and tumor progression [38] (Figure 1).

### 2.2. Role of Cytokine Production in the Pathogenesis of Melanoma

**Figure 2:**
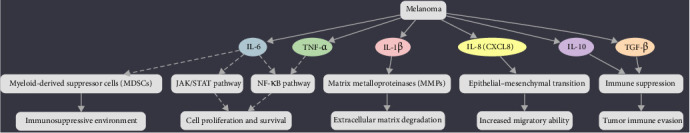
Role of different cytokines in the pathogenesis of melanoma.

Different cytokine types are essential in the development of melanoma, affecting tumor growth, immune responses, and the overall TME [39]. Cytokines can induce inflammation, which subsequently aids in melanoma growth and metastasis. IL-6 and TNF-α enhance the survival and proliferation of melanoma cells [40]. These pro-inflammatory cytokines activate signaling pathways, including JAK/STAT and NF-κB, leading to increased cell proliferation, survival, and resistance to apoptosis [41, 42]. Additionally, these cytokines can stimulate the production of matrix metalloproteinases (MMPs), which degrade the extracellular matrix, thereby promoting melanoma invasion and metastasis [43]. Pro-inflammatory cytokines like IL-1β and IL-8 are critical in promoting melanoma cell growth and invasiveness. They also play a role in breaking down the extracellular matrix, aiding in metastatic spread. Specifically, IL-1β increases the production of adhesion molecules and proteases, which help tumor cells invade and migrate [44]. Meanwhile, IL-8, also known as CXCL8, encourages the epithelial–mesenchymal transition (EMT) in melanoma cells, a process where epithelial cells acquire mesenchymal characteristics and increased migratory ability. This process plays a crucial role in metastasis by enabling tumor cells to break away from the original tumor, infiltrate nearby tissues, and establish secondary tumors in other organs [45]. In melanoma, MDSCs are instrumental in creating an immunosuppressive environment. IL-6, through a STAT3-dependent signaling pathway, increases CCR5 and arginase 1 expression in MDSCs, leading to significant inhibition of CD8+ T-cell activity. In a RET transgenic melanoma mouse model, researchers identified a relationship between IL-6 levels, phosphorylated STAT3, and CCR5 expression in tumor-infiltrating MDSCs. Tumors that overexpressed IL-6 showed slower growth and increased CD8+ T-cell activation in mice. Conversely, blocking IL-6 in transgenic melanoma-bearing mice significantly accelerated tumor progression [46]. Also, it was shown that ILs play a crucial role in the pathogenesis of melanoma by driving inflammation and expanding MDSCs, with IL-1β and the downstream IL-6/STAT3 axis being key contributors to this process [47]. Melanoma cells can secrete immunosuppressive cytokines, including IL-10 and TGF-β, which impair the activity of immune cells such as T cells and NK cells. This suppression allows the tumor to avoid immune detection and destruction [48]. IL-10 can reduce the expression of MHC class II molecules on antigen-presenting cells, hindering the presentation of tumor antigens to T cells [49]. Similarly, TGF-β can diminish the cytotoxic activity of CD8+ T cells and promote the formation of regulatory T cells (Tregs), further weakening the antitumor immune response. This immunosuppressive environment permits melanoma cells to grow without being targeted by the immune system [50] (Figure 2).

## 3. An Overview of IL-2 Cytokine Family

**Figure 3:**
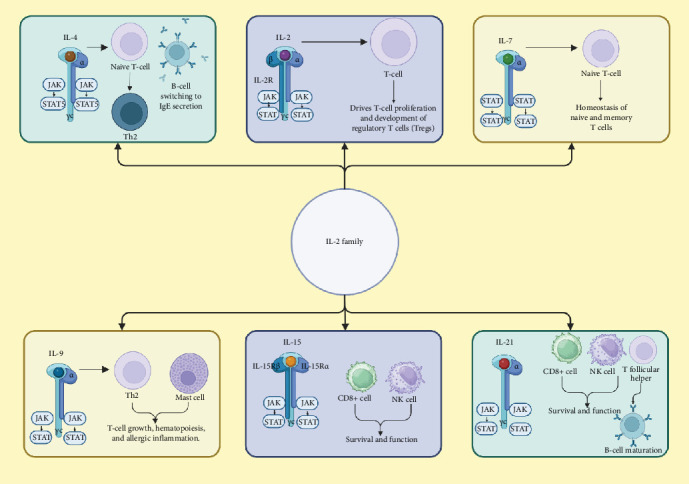
Different types of IL-2 cytokine family, their receptors, and immunomodulatory activities. Created with Biorender.com.

The cytokine family IL-2 within the IL-2 family is one of the important cytokine families that mediate the working of the immune system. IL-2 was discovered in the 1970s as a potent molecule to stimulate T-cell proliferation [51]. As time elapsed, the IL-2 family expanded by adding IL-4, IL-7, IL-9, IL-15, and IL-21, each in its own way modulating the immune system [8]. These cytokines are structurally homologous and use common receptor subunits, specifically the gamma chain (γc), as a signature feature of their signaling pathways. They also serve in collaboration but in very distinct roles within immunity [52]. IL-2 is a potent growth factor for T cells, essential for the expansion of antigen-specific T cells during immune responses [10]. The IL-2 receptor (IL-2R) is one of the most extensively researched receptors in the IL-2 family. It comprises three subunits: α (CD25), β (CD122), and the common γc (CD132) [53, 54]. This composition allows IL-2R to bind IL-2 with specific affinities, which vary based on the subunit configuration. The combination of all three subunits results in a high-affinity IL-2R complex, which is crucial for potent biological activities like the clonal expansion of antigen-specific T cells during an immune response[55]. When IL-2 binds to its receptor complex (IL-2R), it induces conformational changes that activate intracellular signaling pathways, primarily the JAK-STAT pathway. This receptor initiates the activation of Janus kinases, JAK1 and JAK3, which subsequently phosphorylate STAT proteins. Once phosphorylated, these STAT proteins dimerize and translocate to the nucleus, where they regulate gene expression essential for T-cell proliferation, survival, and differentiation [56]. Phosphorylated STAT proteins form dimers and translocate to the nucleus, where they trigger gene expression necessary for T-cell proliferation, survival, and differentiation [57]. Moreover, IL-2R signaling is vital not only for effective T-cell activation but also for the ongoing development and maintenance of Tregs, which are crucial in preventing severe autoimmunity [58]. IL-4 primarily drives the differentiation of naive T cells into T-helper 2 (Th2) cells, which are key regulators of humoral immunity in response to extracellular pathogens [59]. IL-4 signaling through the IL-4R (comprised of the subunits IL-4Rα and γc) activates the STAT6 pathway, which induces gene expression important for B-cell class switching to IgE for allergic responses and antiparasitic defense [60]. IL-7 is absolutely required for the development of T cells and B cells and is especially critical early in lymphopoiesis. IL-7 acts through its heterodimer receptor, IL-7R, which is composed of IL-7Rα and γc, to activate the JAK-STAT signaling pathway. The main function of IL-7 is in the homeostasis of naïve and memory T cells for the preservation of a healthy adaptive immune system [61]. Initially recognized as a T-cell growth factor, IL-9 has a much wider range of functions, including regulating hematopoiesis and contributing to the immune response against helminth infections. Its receptor, IL-9R, contains α and γc subunits. IL-9 has both pro-survival and proliferative effects on mast cells, in addition to enhancing the production of Th2 cytokines and chemokines, thereby amplifying the functions of these cells in allergic inflammation and asthma [62]. IL-15 shares many biological functions with IL-2, particularly in supporting the development and maintenance of NK cells and memory CD8+ T cells. It signals through a receptor complex consisting of IL-15Rα, IL-2/15Rβ (shared with IL-2Rβ), and γc. IL-15 is essential for the survival and proliferation of these cells, making it a key player in the immune system's defense against viral infections and tumors [63]. The most recently identified member of the IL-2 cytokine family is IL-21, which has diverse roles in immune responses. It signals through a receptor made up of IL-21Rα and γc, impacting the activity of T cells, B cells, NK cells, and DCs. IL-21 is crucial for promoting the differentiation of T follicular helper (Tfh) cells, which aid in B-cell maturation and antibody production. Additionally, IL-21 enhances cytotoxic T-cell responses and supports the function of NK cells [64] (Figure 3).

The common γc cytokine family—which includes ILs IL-2, IL-4, IL-7, IL-9, IL-15, and IL-21—signals through receptor complexes containing the γc subunit, activating key intracellular pathways such as JAK/STAT, PI3K/Akt, and MAPK/ERK [65]. Upon receptor engagement, JAK1 and JAK3 kinases are activated, leading to the phosphorylation of specific STAT proteins; IL-2, IL-7, IL-9, and IL-15 primarily activate STAT5; IL-4 predominantly activates STAT6; and IL-21 activates STAT3, with some overlap among these cytokines. These STAT proteins regulate gene expression critical for immune cell development, survival, and function. Additionally, the PI3K/Akt pathway promotes cell survival and metabolism, while the MAPK/ERK pathway influences cell proliferation and differentiation. The specific downstream effects depend on the cytokine involved, the receptor composition, and the cellular context, allowing for precise modulation of immune responses [9, 66].

## 4. Regulatory Factors of IL-2 Cytokine Family

IL-2 expression is intricately regulated through multiple mechanisms to ensure appropriate immune responses. Activation of the TCR initiates signaling pathways that activate transcription factors such as NFAT, AP-1, and NF-κB, which bind to the IL-2 promoter to induce transcription [67]. Costimulatory signals, particularly through CD28 engagement, are essential for full IL-2 gene activation [68]. The chromatin state of the IL-2 locus also plays a critical role; in resting T cells, the promoter region is associated with repressive histone modifications and low acetylation, limiting transcription factor access. Upon activation, histone acetylation increases, enhancing chromatin accessibility and facilitating transcription [69]. Negative feedback mechanisms also exist; IL-2 induces the expression of BLIMP-1, which in turn represses IL-2 transcription, and regulatory T cells expressing FOXP3 can suppress IL-2 production, maintaining immune homeostasis [70]. IL-4 expression is regulated by a complex interplay of transcription factors, epigenetic modifications, and cell-type-specific mechanisms. The transcription factors GATA3 and c-Maf are central to IL-4 regulation; GATA3 is essential for Th2 differentiation and IL-4 expression, while c-Maf directly transactivates the IL-4 gene [71]. Epigenetically, chromatin remodeling, including histone modifications and DNA methylation, modulates the accessibility of the IL-4 locus, influencing its transcriptional activity [72]. The IL-4 gene can also exhibit monoallelic or biallelic expression patterns, which may be influenced by the strength and duration of TCR signaling [73]. Furthermore, IL-4 production is not limited to Th2 cells; innate immune cells like basophils, mast cells, and eosinophils can produce IL-4 upon activation, contributing to the cytokine secretion [74]. The expression of the IL-7 receptor alpha chain (IL-7Rα or CD127) on T cells is tightly controlled; IL-7 binding leads to transient downregulation of IL-7Rα, serving as a feedback mechanism to modulate T-cell responsiveness and maintain homeostasis. Additionally, transcription factors such as PU.1 and GABP have been identified as regulators of IL-7Rα gene expression, highlighting the role of transcriptional control in IL-7 signaling pathways [75]. Furthermore, the IL-7 signaling cascade involves the activation of the JAK/STAT pathway, particularly JAK1 and JAK3 kinases, which are essential for transmitting survival and proliferation signals to lymphocytes [76]. IL-9 expression is regulated by transcription factors such as PU.1 and IRF4. The CNS-25 enhancer upstream of the IL9 gene plays a crucial role by recruiting these factors and facilitating chromatin looping to promote transcription [77]. Cytokines like IL-4, IL-33, and TGF-β induce IL-9 production, while IL-25 enhances its expression via the IL-17RB receptor [78]. Conversely, IFN-γ and IL-23 can suppress IL-9 synthesis [79]. IL-15′s expression is tightly regulated due to its potent immunostimulatory effects. Transcription factors such as IRF-1, RUNX3, T-bet, and Eomesodermin (Eomes) are involved in its regulation. IL-15 is presented in trans-to-target cells through its high-affinity receptor IL-15Rα, forming a complex that is crucial for its stability and function [80]. IL-21 expression is regulated by transcription factors, including c-Maf, Bcl-6, and c-Rel. Epigenetic regulation involves long-range chromatin interactions, with conserved noncoding sequences facilitating transcription. Cytokines, including IL-6, can induce IL-21 production, while T-bet acts as a repressor in certain contexts [81].

## 5. The Role of IL-2 Cytokine Family in the Pathogenesis and Treatment of Various Cancers

Originally identified for its ability to promote the growth and differentiation of T cells, IL-2 is now known to have broader significance in immune system regulation. Extensive research has explored its role in cancer development and therapy, highlighting its dual function in enhancing immune responses and maintaining immune tolerance [14]. IL-2 is pivotal in the process of immunoediting, which describes the dynamic interaction between the immune system and tumor cells. Immunoediting occurs in three stages: elimination, equilibrium, and escape. During the elimination phase, IL-2 boosts the proliferation and activation of cytotoxic T cells and NK cells, empowering them to detect and destroy cancer cells [14, 82]. Levels of soluble IL-2R (sIL-2R) are frequently elevated in lymphoma, offering valuable information about disease progression and prognosis. High sIL-2R levels can indicate T-cell activation, potentially signaling an antitumor response in malignant diseases if the sIL-2R is primarily produced by effector T cells [83]. In T-cell lymphomas, elevated sIL-2R levels are associated with shorter median survival times, suggesting a more aggressive disease and higher tumor load. This has prompted the proposal to incorporate sIL-2R levels into prognostic indices for risk-adapted therapeutic strategies. Similarly, in B-cell lymphomas, including follicular lymphoma and diffuse large B-cell lymphoma, high sIL-2R levels are linked to greater tumor burden and worse progression-free survival (PFS) [84] IL-2 plays a role in the progression of cervical cancer by triggering metabolic reprograming through the JAK/STAT pathway, specifically by activating STAT5. This activation boosts glycolysis and energy production, which supports the rapid proliferation of cervical cancer cells [85]. IL-2, particularly in the form of IL2-MSA (IL-2 fused with murine serum albumin), has been found to boost the binding of IL12-MSA to tumor-specific CD8+ T cells. Coadministration of IL12-MSA and IL2-MSA encourages the differentiation of these effector T cells, reduces the population of tumor-infiltrating regulatory T cells (CD4+ Tregs), and enhances survival outcomes in mice with lung tumors [86]. A study examined the use of a cyclodextrin (CD) nanoplex system to simultaneously deliver the anticancer drug 5-fluorouracil (5-FU) and IL-2 for colorectal cancer treatment, combining chemotherapy and immunotherapy. Researchers developed a nanocapsule containing 40% 5-FU and 99.8% IL-2. This nanoscale delivery system improves the anticancer effectiveness of 5-FU and IL-2 while minimizing toxicity, representing a promising strategy for treating colorectal cancer [87]. A study investigated IL-2 levels in breast cancer patients compared to healthy women. Additionally, it compared IL-2 levels between HER-2 positive and HER-2 negative patients, ER/PR positive and ER/PR negative patients, and across different malignancy grades of breast cancer. The findings revealed that IL-2 levels are elevated in breast cancer patients, particularly those with HER-2-positive expression [88]. IL-4 facilitates Th2-cell differentiation while suppressing Th1 cells. Within cancer microenvironments, IL-4 influences pathways that govern cancer cell survival, proliferation, and metastasis. It also affects cancer cell migration, invasion, and metabolic processes [89]. IL-4Rα, primarily expressed in non-hematopoietic cells, is recognized as the main target for IL-4. The IL-4 receptor is thought to be overexpressed and significant in pancreatic cancer. Its roles include inducing tumor formation, promoting cancer cell proliferation, and contributing to apoptotic resistance [90]. Single-cell RNA sequencing of non-small cell lung cancer (NSCLC) lesions in humans and mice revealed that in both species, IL-4 is likely the main driver of the tumor-infiltrating monocyte-derived macrophage phenotype [91]. IL-7 has been found to inhibit the progression of colon cancer. Additionally, IL-7 levels are associated with OS and pathological stage in patients. Clinical data analysis confirms that IL-7 is a crucial factor in inhibiting colon cancer progression [92]. Research has demonstrated that IL-7 can boost both the quantity and activity of tumor-infiltrating lymphocytes in colorectal cancer patients. A clinical trial revealed that combining IL-7 with chemotherapy markedly improved the survival rate of patients with advanced colorectal cancer [93]. IL-7 has been widely researched for lymphoma treatment. One study discovered that IL-7 can increase the number of CD4+ and CD8+ T cells and enhance their function in lymphoma patients. Combining IL-7 with chemotherapy or immunotherapy has been proposed as a potential strategy to improve treatment response and survival rates in these patients [94]. Various tumor cell-related and TME-related factors can impact IL-9 production, affecting anti-PD-1 treatment *efficacy*. TGF-β and IL-4 notably induce IL-9 expression in CD4 T cells, but prolonged high TGF-β levels reduce IL-9 in tumors. Continuous TGF-β exposure suppresses BFAR expression, creating a feedback loop that limits TGF-β signaling, reducing Smad2/3 phosphorylation and IL-9 production under TH9 conditions. TGF-β can also hinder PD-1/PD-L1 blockade response. Blocking TGF-β in the TME improves PD-1 blockade response by increasing IL-9 levels and TH9-cell infiltration in tumors, an effect not seen in other CD4 T cells. Blocking IL-9 reverses the benefits of TGF-β neutralization on anti-PD-1 treatment, indicating IL-9 suppression partly mediates TGF-β's adverse effect on PD-1 responsiveness [95]. IL-9 is a “double-edged sword” in tumor immunity, promoting both tumor growth and antitumor immunity. In solid tumors like melanoma, IL-9 supports antitumor responses by inducing apoptosis and activating immune cells. Th9 cells, which secrete IL-9, enhance CTL activity and reduce tumor growth. Blocking IL-9 reverses these benefits, underscoring its antitumor role. However, in metastatic lung cancer, IL-9 promotes metastasis [96, 97]. In gastric cancer, IL-9 inhibits tumor cell growth and correlates with better patient outcomes and higher CD8+ T-cell activity [98]. Similar antitumor effects are seen in colon [99] and breast cancers [100]. In hematologic malignancies, IL-9 promotes tumor growth by activating JAK/STAT pathways and preventing apoptosis. Elevated IL-9 levels in lymphomas correlate with poor prognosis. Blocking IL-9 inhibits tumor growth in lymphoma models. In T-cell cancers, IL-9 enhances leukemic cell survival and proliferation [101]. IL-15 is emerging as a highly promising target for cancer immunotherapy. Preclinical studies have shown that IL-15 agonists can substantially inhibit tumor growth and possess anti-metastatic effects. These findings have led to ongoing clinical trials to further evaluate their potential [102]. IL-21, a type 1 cytokine, with a 4α-helix bundle structure that has demonstrated significant potential in cancer immunotherapy. It enhances antitumor responses of immune cells and aids in the differentiation of memory T cells [103]. It was found that attaching IL-21 to an anti-PD-1 antibody (PD-1Ab21) boosts the generation and proliferation of memory stem T cells (TSCM). In tumor-bearing mice, PD-1Ab21 outperforms the combination of PD-1 blockade with IL-21 infusion by enhancing antitumor effects, increasing TSCM, and expanding tumor-specific CD8+ T cells. This approach enhances immune checkpoint therapy by targeting cytokines directly to tumor-reactive T cells [104] (Table 1).

## 6. Role of IL-2 Family in the Pathogenesis of Melanoma: Focusing on Molecular Mechanisms

The IL-2 cytokine family is integral to the development of melanoma, impacting both tumor growth and immune regulation. This part explores the molecular pathways through which IL-2 family members influence melanoma advancement, emphasizing their roles in interacting with immune cells and the surrounding TME. Research analyzing IL-2 levels in melanoma patients found that serum sIL-2R concentrations were notably higher than in healthy individuals, with elevated levels consistently present throughout all stages of melanoma. Particularly, elevated sIL-2R levels were linked to a poorer prognosis, especially in cases where the levels surpassed 529 U/ml. Additionally, sICAM-1 concentrations were found to be increased, notably during metastatic phases. The high levels of sIL-2R may indicate a risk factor for the malignant progression of melanoma, suggesting that these serum markers could be valuable in monitoring disease progression and prognosis [105]. Tumor-derived active TGF-β greatly diminishes the anticancer effects mediated by IL-2. Studies have demonstrated that TGF-β obstructs the phosphorylation and activation of key components in the JAK/STAT signaling pathway, which function downstream of the IL-2R [106]. In melanoma, the chimeric protein soluble extracellular domain of TGF-βR II (FIST), which merges IL-2 with the soluble extracellular domain of TGF-β receptor II, functions as a decoy receptor for TGF-β. By sequestering TGF-β, FIST negates its immunosuppressive effects, thereby allowing IL-2 to bolster immune activation. This mechanism results in the hyperactivation of STAT1 within immune cells, increased production of proinflammatory cytokines, and a robust antitumor response, ultimately suppressing melanoma growth in immunocompetent mouse models [107]. Blocking PD-1 in melanoma enhances the antitumor activity of CD8+ T cells. However, it can also cause a rise in immunosuppressive Treg cells within the tumor, potentially diminishing the therapy's overall impact. Studies have found that this rise in tumor-Tregs is not a direct consequence of PD-1 signaling inhibition in Tregs. Instead, it occurs indirectly due to the activity of activated CD8+ T cells. These CD8+ T cells release IL-2, which promotes the expression of the anti-apoptotic protein ICOS on tumor-Tregs, facilitating their accumulation. Suppressing ICOS signaling can help counteract the negative impact of Treg cells, thereby improving the effectiveness of PD-1 blockade therapy. Disrupting the interaction between CD8+ T cells and Tregs within the TME may present a promising approach to enhancing the success of PD-1 immunotherapy [108].

In the development of melanoma, IL-4 emerges as a crucial cytokine for assessing metastatic risk. Increased levels of IL-4, along with GM-CSF and dermcidin (DCD), have been recognized as significant biomarkers in early-stage melanoma. IL-4 contributes to the disease by affecting immune responses that may promote tumor progression and metastasis, thereby making it an important factor in prognostic evaluations for estimating the likelihood of metastasis in melanoma patients [109]. IL-4 significantly contributes to inhibiting melanoma progression, as evidenced by experiments involving IL-4-overexpressing transgenic mice compared to non-transgenic mice. The IL-4 transgenic mice displayed smaller tumor volumes and weights and showed enhanced activation of the STAT6 pathway. This was demonstrated by increased DNA binding activity, phosphorylation of STAT6, and higher expression levels of IL-4, IL-4Rα, and p21 in their tumor tissues. Additionally, tumors from these IL-4 mice had elevated levels of apoptotic proteins such as cleaved caspases, Bax, p53, and p21, while exhibiting decreased levels of the survival protein Bcl-2. In vitro experiments further indicated that IL-4 overexpression hampers melanoma cell proliferation through p21-mediated STAT6 pathway activation, leading to upregulated expression of apoptotic proteins. When p21 was knocked down, melanoma cell growth suppression was reversed, accompanied by decreased IL-4 expression and reduced STAT6 activation. These results imply that IL-4 suppresses melanoma development by inducing apoptosis through the activation of the p21-STAT6 pathway [110]. Higher plasma levels of IL-2 and IL-4 have been found to significantly correlate with reduced pigmentation in melanoma patients, especially those with acral melanoma. This finding suggests that IL-2 and IL-4, along with other cytokines, including IL-5, IL-10, IL-12, IL-13, GM-CSF, IFN-γ, and TNF-α, may play a role in regulating melanoma pigmentation [111].

In melanoma patients, lower levels of IL-7 are associated with CD8+ T-cell exhaustion, as shown by decreased soluble CD127 (sCD127) levels and altered CD127 expression. IL-7 can enhance the cytotoxic activity of CD8+ T cells and increase sCD127 release, primarily via the PI3K signaling pathway. Nonetheless, melanoma patients display reduced CD8+ T-cell cytotoxicity, suggesting that insufficient IL-7 may contribute to dysfunctional CD8+ T-cell responses and limit effective antitumor immunity [112]. Treatment with BRAFV600E or MEK1/2 inhibitors significantly reduced IL-7 secretion and lowered pERK and pMEK levels in BRAFV600E-mutated melanoma cell lines. BRAF wild-type melanomas showed minimal changes. As a result, it was suggested that MAPK pathway inhibition can alter the immune profile of BRAFV600E melanoma cells, potentially informing combination therapies with BRAF/MEK inhibitors and immunotherapy [113]. IL-7 is linked to disease progression in melanoma, with lower serum levels observed in patients, especially those with BRAF mutations. It was found that, although melanoma patients had increased levels of various cytokines and growth factors, IL-7 was reduced compared to healthy controls. Additionally, lower IL-7 levels were associated with specific genetic mutations and the overall cytokine profile in melanoma. This suggests that IL-7 may contribute to melanoma development and could serve as a valuable marker for tumor progression and patient prognosis [114].

Research has shown that IL-9, when expressed on B16F10 melanoma cells as either a secretory (sIL-9) or membrane-bound form (mbIL-9), significantly reduced lung metastases in mice. IL-9 promoted the infiltration of immune cells, particularly M1 macrophages, into the lungs and encouraged peritoneal and RAW264.7 macrophages to shift toward an M1 phenotype, enhancing their antitumor activity. These findings suggest that IL-9 may inhibit metastasis by driving M1 macrophage polarization and activation, thereby improving their ability to combat melanoma cells [115]. IL-9 boosts melanoma progression by improving the metabolism and survival of Tc9 cells, which secrete IL-9. Tc9 cells have lower lipid peroxidation and higher antitumor activity than Tc1 cells due to IL-9′s activation of STAT3 and increased fatty acid oxidation. This protection against ferroptosis in the tumor environment highlights the potential of targeting the IL-9/STAT3/fatty acid oxidation pathway to enhance T cell –based therapies for melanoma [116].

## 7. Targeting IL-2 Cytokine Family Receptors in Melanoma

Targeting IL-2 cytokine family receptors in melanoma represents a promising approach to enhancing immune responses against this aggressive SC. Both preclinical studies and clinical trials have explored the efficacy and mechanisms of these targeted therapies, aiming to improve treatment outcomes and overcome resistance in melanoma patients.

### 7.1. Advances in IL-2 Family–Based Immunotherapies for Melanoma: Focus on Preclinical Studies

An engineered IL-2–based cytokine, such as nemvaleukin, appears promising for melanoma treatment. In preclinical studies using humanized mice models with patient-derived tumor cells and immune cells, nemvaleukin and anti-PD-1 therapies produced antitumor responses specific to individual patients, closely reflecting clinical outcomes in melanoma. Nemvaleukin increased tumor-infiltrating CD4+ and CD8+ T cells, promoted the expansion of non-regulatory T-cell populations, and significantly slowed tumor growth compared to controls and recombinant human IL-2 (rhIL-2) treatment. These findings indicate that nemvaleukin might be a valuable new immunotherapy option, potentially improving treatment outcomes for melanoma patients with diverse responses to existing therapies [117]. In adoptive T-cell therapy (ACT) for melanoma, combining total body irradiation (TBI) and IL-2 aims to improve the persistence and function of transferred tumor-specific T cells. TBI removes immunosuppressive cells, while IL-2 supports T-cell survival. However, this combination does not yield the expected benefits; it increases T-cell numbers in tissues but reduces their activation compared to TBI alone. Additionally, it decreases DCs in the tumor, which are crucial for T-cell activation. Therefore, while both TBI and IL-2 enhance ACT individually, their combination may have complex and potentially harmful effects on the immune environment in tumors [118]. IL-2 and IL-15 are cytokines known for their immune-boosting properties, which are significant in melanoma treatment, particularly in modulating immune responses in regional lymph nodes (LNs), a common site for early melanoma metastasis. These cytokines enhance the expression of the activating receptor NKG2D and the inhibitory receptors CD158a and CD158b on CD8+ T cells, NKT-like cells, and NK-cell subsets in the LNs of melanoma patients. These alterations boost the antitumor cytotoxicity of NK cells and increase the overall immune capability of these lymphocyte subsets. By upregulating NKG2D receptor expression and activating immune cells, IL-2 and IL-15 enhance the antitumor response in LNs, potentially improving local immune interventions and aiding in tumor control [119]. IL-2 has been used in gene therapy via electroporation to introduce therapeutic genes into cells. In a study, combining gene electrotransfer of IL-2 and IL-12 at the tumor site significantly slowed tumor growth and led to complete regression in 71% of cases in a murine B16.F10 melanoma model. This approach increased DCs and M1 macrophages in tumors, activated proinflammatory signals, and attracted CD4+ and CD8+ T-lymphocytes. It also promoted immune memory, enhancing the body's ability to fight melanoma. Thus, the combined gene electrotransfer of IL-2 and IL-12 showed strong antitumor effects and potential long-term protection [120]. Celastrol (CEL), an IL-2/CD25 binding inhibitor, impacts melanoma treatment by blocking IL-2 from interacting with the CD25 receptor, thereby disrupting IL-2 signaling in T cells. In murine models, CEL reduced B16F10 melanoma growth by increasing CD8+ T cells but was ineffective in T cell–deficient mice, indicating its reliance on T-cell activity. Combining CEL with a TNFR2 antagonist improved results by boosting the ratio of CD8+ T cells to Tregs and decreasing Foxp3 expression. CEL's ability to inhibit IL-2/CD25 binding and its synergistic effect with other treatments highlight its potential for use in combination therapies for melanoma [121]. LOAd732 is a novel oncolytic adenovirus engineered to boost antitumor immunity. It carries three important immunostimulatory transgenes—CD40L, 4-1BBL, and IL-2—that activate DCs and enhance immune responses against melanoma. Research shows that LOAd732 infects melanoma cells and DCs while maintaining its oncolytic activity. The virus effectively matures DCs, increasing their expression of costimulatory molecules and cytokines essential for T and NK-cell activation. Remarkably, LOAd732-matured DCs continue to stimulate antigen-specific T cells even in the presence of immunosuppressive cytokines such as TGF-β1 and IL-10. These results indicate that LOAd732, by activating DCs and improving T- and NK-cell responses despite a challenging TME, is a promising approach for cancer immunotherapy [122].

IL-4 enhances melanoma progression by interacting with preadipocytes. Coculturing B16 melanoma cells with 3T3L1 preadipocytes increased cell growth and levels of metastasis-related proteins. This effect, driven by exosomal interactions, was reduced by the exosome inhibitor GW4869. Coculturing with macrophages also increased M2 markers, which were diminished by GW4869. These findings highlight the role of preadipocytes and exosomes in melanoma advancement, with IL-4 contributing to M2 macrophage polarization [123].

IL-9 enhances the effectiveness of T cells in melanoma treatment through engineered chimeric receptors. By fusing an IL-2R extracellular domain with an IL-9 receptor intracellular domain, IL-2 mimics IL-9 signaling. T cells with this chimeric receptor (o9R T cells) show superior antitumor activity, activating key signaling pathways and acquiring beneficial T-cell traits. In mouse models, o9R T cells were more effective than conventional T cells and worked well without prior lymphodepletion. Thus, IL-9 signaling through this chimeric receptor improves T-cell function and antitumor response in solid tumors like melanoma [124].

The novel fusion protein SON-1210, combining IL-15 and IL-12 with a human albumin-binding domain, enhances melanoma treatment by improving pharmacokinetics and targeting. In B16F10 melanoma mouse models, SON-1210 demonstrated superior efficacy compared to native cytokines, significantly reducing tumor growth and enhancing immune responses, including elevated IFNγ levels and increased Th1 and CTL cell counts. It also shifted macrophages to a pro-inflammatory M1 phenotype and decreased Treg cells. Safety and prolonged activity were confirmed in nonhuman primate studies, suggesting that SON-1210 is a promising candidate for solid tumor immunotherapy with high effectiveness and minimal toxicity [125]. Administering IL-15 fused with IL-15Rα (IL-15/IL-15Rα) directly into tumors boosts tumor cell destruction and expands NK and CD8+ T cells. This treatment also activates NK and CD4+ T cells, with NK cells being crucial for attacking tumors. When IL-15/IL-15Rα is combined with a PD-L1-specific antibody (K2-Fc), it further enhances tumor cell killing by boosting NK-cell activation independently of T cells. These results indicate that IL-15/IL-15Rα gene therapy could significantly enhance immune responses and improve the effectiveness of PD-L1 blockade therapies for melanoma [126]. Intranasal administration of Neospora caninum modified to produce human IL-15 fused with IL-15Rα shows promise in treating lung metastases from melanoma. In a B16F10 lung metastasis mouse model, this method significantly decreased metastasis coverage in the lungs (0.08% with IL-15/IL-15Rα-expressing N. caninum compared to 4.4% with wild-type and 36% in untreated controls). The treatment also boosted immune cell levels and shifted macrophages to a more antitumor M1 phenotype. These findings indicate that this strategy could be a promising, non-invasive treatment for metastatic lung cancer[127] (Table 2).

### 7.2. Recent Clinical Trials on IL-2 Cytokine Family–Based Therapeutics for Treatment of Melanoma

Recent Phase II and III trials have evaluated various combinations of IL-2 therapies for melanoma. For instance, the Phase II trial (NCT02581930) investigated the use of Ibrutinib combined with IL-2 inducible kinase but found no significant antitumor responses or clinical benefits compared to conventional therapies like PD-1 and MAPK inhibitors, with a PFS of only 1.3 months [128]. Conversely, another Phase II trial (NCT01416831) assessed the combination of stereotactic body radiotherapy (SBRT) and IL-2 and found improved overall response rates (ORRs) and disease control rates (DCRs) compared to IL-2 monotherapy, though progression-free and OS rates were not significantly different [129]. Studies focusing on high-dose IL-2 have shown promising results. For instance, a retrospective cohort study highlighted that high-dose IL-2 (600,000–720,000 U/kg/dose) led to a prolonged PFS of over 5 years for patients who received no subsequent systemic therapy [130]. Another trial evaluated the combination of high-dose IL-2 with radiotherapy, reporting a partial response rate of 15.7% and a DCR of 52.6% after a median follow-up of 39.2 months. Notably, this study observed a strong immunological response with increased levels of specific cytokines and T cells [131]. Intralesional IL-2 therapies have been evaluated in various studies, showing variable results. One study found a 44.6% complete response rate and a 65.5% durable response rate in patients with in-transit melanoma, with a median time to recurrence of 10.5 months [132]. Another study combining systemic PD-1 inhibitors with intralesional IL-2 (PROLEUKIN) showed a 74% response rate in locoregional metastases and improved OS, particularly in patients without active distant metastases [133]. Some studies highlight the potential benefits of combining IL-2 therapies with other treatments, such as PD-1 inhibitors or radiotherapy, the results are mixed. For example, a Phase Ib trial (NCT02748564) combining pembrolizumab with high-dose IL-2 reported a partial response in only 11% of patients, indicating that the maximum tolerated dose (MTD) was not reached and suggesting limited effectiveness of this combination compared to standard treatments [134]. Overall, recent studies reflect a spectrum of outcomes for IL-2–based therapies in melanoma treatment. While high-dose IL-2 and its combinations with other modalities have shown some promise, the efficacy varies, and further research is needed to optimize treatment strategies and improve patient outcomes. A Phase I/Ib clinical trial (NCT02452268) evaluated NIZ985, a recombinant form of IL-15 and its receptor α, as a single agent and in combination with the anti-PD-1 antibody spartalizumab in melanoma patients. The study focused on tumors that were resistant to standard immune checkpoint therapies. The results demonstrated antitumor effects, with DCRs reaching 45% in PD-1-sensitive tumors and 20% in those resistant to PD-1 inhibition. Patients treated with NIZ985 alone achieved stable disease, while the combination with spartalizumab led to partial responses, highlighting the potential of this combination in treating difficult melanoma cases [135]. Adverse events associated with immunotherapies such as high-dose IL-2, PD-1 inhibitors, and combination treatments (e.g., Ibrutinib, SBRT, NIZ985 with spartalizumab) are diverse and span multiple organ systems. Common systemic symptoms include fatigue, fever, chills, rigors, flu-like illness, myalgia, arthralgia, headache, dizziness, and weight loss. Gastrointestinal effects such as nausea, vomiting, diarrhea, constipation, anorexia, abdominal pain, and dysgeusia are frequently observed, along with metabolic abnormalities like hypoalbuminemia, hyponatremia, and adrenal insufficiency. Dermatologic and immune-related adverse events include rash, pruritus, vitiligo, and immune-mediated hypothyroidism. Cardiovascular complications such as hypotension (requiring vasopressors) and premature ventricular contractions, as well as pulmonary issues like dyspnea and capillary leak syndrome, have also been reported. Neurological symptoms range from dizziness and peripheral neuropathy to mental status changes and, in rare cases, normal pressure hydrocephalus. Musculoskeletal complaints (arthralgia, arthritis, myalgia), hematologic issues (anemia, lymphocyte count decrease), and elevated liver enzymes further highlight the systemic nature of toxicity. These effects are noted across various dosing regimens, including intralesional IL-2 (3–18 MIU), high-dose IL-2 (600,000–720,000 U/kg), PD-1 inhibitors (e.g., pembrolizumab), and novel agents like NIZ985, underlining the importance of vigilant monitoring during treatment (Table 3).

## 8. Conclusion and Future Perspectives

The exploration of the IL-2 cytokine family in melanoma treatment highlights its significant role in modulating immune responses and providing potential therapeutic targets. The IL-2 family, encompassing IL-2, IL-4, IL-7, IL-9, IL-15, and IL-21, plays diverse roles in immune system regulation. These cytokines leverage common receptor subunits, particularly the γc, to exert their effects, which range from T-cell proliferation and differentiation to the regulation of humoral and cellular immunity. IL-2 itself is a potent growth factor for T cells, crucial for their clonal expansion during immune responses. The IL-2R complex, composed of the α, β, and γc subunits, facilitates this process through the JAK-STAT signaling pathway. Similarly, IL-4, IL-7, IL-9, IL-15, and IL-21, despite their structural homology, each play distinct roles, such as directing T-cell differentiation, supporting lymphocyte development, and enhancing antitumor activity. The therapeutic potential of targeting the IL-2 cytokine family in melanoma has been demonstrated through various preclinical and clinical studies. Engineered IL-2 family cytokines and recombinant forms of cytokines have shown promise in enhancing antitumor responses by increasing tumor-infiltrating T cells and promoting the expansion of non-regulatory T-cell populations. Moreover, combination therapies involving IL-2 and other modalities, such as PD-1 inhibitors and radiotherapy, have yielded variable but promising results, highlighting the complexity and potential of cytokine-based treatments in melanoma.

The role of the IL-2 cytokine family in melanoma pathogenesis and treatment is multifaceted, with evidence supporting both pro- and antitumor effects. These seemingly contradictory findings highlight the complex immunoregulatory functions of this cytokine family and underscore the importance of context—particularly the TME, immune cell interactions, cytokine concentrations, and genetic mutations. Some studies suggest a paradoxical contribution of IL-2 to melanoma progression. Elevated serum levels of sIL-2R are consistently observed in melanoma patients and are correlated with poor prognosis, particularly when levels exceed 529 U/ml. These elevated levels may reflect heightened immune activation but also suggest a potential mechanism by which melanoma evades immune surveillance through immune exhaustion or dysregulation. Furthermore, IL-2 release by activated CD8+ T cells can inadvertently promote the survival of tumor-infiltrating Tregs via the upregulation of ICOS, which dampens the overall effectiveness of immunotherapies like PD-1 blockade. Conversely, IL-2 has well-established immunostimulatory properties. Engineered IL-2 variants such as nemvaleukin have shown promising preclinical results by selectively expanding tumor-infiltrating effector T cells while avoiding regulatory T-cell expansion, thereby enhancing antitumor immunity. Gene therapy approaches utilizing IL-2, either alone or in combination with IL-12, significantly suppress melanoma growth by promoting immune memory, DC activation, and T-cell infiltration. This duality is not unique to IL-2 alone but extends to other family members such as IL-4, IL-7, IL-9, and IL-15, which also show context-dependent behavior. These findings suggest that the IL-2 cytokine family cannot be universally categorized as either pro- or antitumor. Instead, their role is heavily influenced by the molecular and cellular context. Therapeutic strategies must, therefore, be tailored to exploit the beneficial immune-activating effects of IL-2 while minimizing its capacity to sustain immunosuppressive elements like Tregs.

However, the therapeutic manipulation of IL-2 family cytokines is not without risk. These agents, particularly when used at high doses or in combination regimens, can cause immune overactivation, leading to severe side effects such as cytokine storms and autoimmune reactions. The overstimulation of T cells and NK cells can result in systemic inflammation and organ damage. Moreover, by reducing the number of regulatory T cells and enhancing effector responses, these therapies may tip the balance toward autoimmunity. Thus, careful patient selection, dosing strategies, and ongoing monitoring are essential to maximize the therapeutic benefits while minimizing potential harm. A deeper understanding of the immune landscape in melanoma and the nuanced roles of cytokine signaling will be crucial for the safe and effective application of these therapies.

Future research should continue to focus on optimizing the therapeutic applications of the IL-2 cytokine family in melanoma. While combination therapies have shown promise, the interactions between different treatments need to be better understood. Investigating the synergistic effects of IL-2 family cytokines with ICIs, targeted therapies, and conventional treatments could yield more effective strategies for melanoma management. Personalized approaches that consider individual patient profiles, including genetic mutations and immune status, are essential. Tailoring cytokine-based therapies to the specific immune landscape of each patient could enhance treatment efficacy and minimize adverse effects. Advances in cytokine engineering, such as the development of more potent and selective cytokine variants, could improve therapeutic outcomes. Comprehensive mechanistic studies are required to clarify the specific signaling pathways and molecular interactions driven by IL-2 family cytokines in melanoma. Understanding these mechanisms can inform the design of more effective therapeutic interventions. Identifying reliable biomarkers for predicting response to cytokine-based therapies is crucial. Biomarkers could guide treatment decisions, monitor therapeutic efficacy, and predict potential resistance, thereby improving patient outcomes. Resistance to cytokine-based therapies remains a significant challenge. Research should focus on identifying the mechanisms of resistance and developing strategies to overcome them, such as combination treatments that target multiple pathways involved in melanoma progression. In conclusion, the IL-2 cytokine family represents a promising avenue for melanoma treatment. Ongoing research and clinical advancements in this field hold the promise of greatly enhancing treatment outcomes for patients battling this aggressive form of SC. By addressing the challenges and leveraging the opportunities presented by cytokine-based therapies, future advancements could lead to more effective and personalized treatments for melanoma.

## Figures and Tables

**Table 1:** Summary of the roles of various interleukins in cancer, detailing their mechanisms, effects, and associated cancer types.

Interleukin (IL)	Role in cancer	Molecular mechanism	Outcomes	Cancer types
IL-2	Immune response regulation, cancer therapy	Enhances T cell and NK cell proliferation; involved in immunoediting (elimination, equilibrium, escape); promotes glycolysis via JAK/STAT pathway	Elevated sIL-2R levels indicate disease progression in lymphomas; enhanced efficacy of IL12-MSA in lung tumors; improved survival with combined 5-FU and IL-2 in colorectal cancer	Lymphomas, cervical cancer, lung cancer, colorectal cancer
IL-4	Cancer progression	Facilitates Th2 differentiation; influences cancer cell survival, proliferation, and metastasis; impacts migration and invasion	Overexpressed in pancreatic cancer; drives macrophage phenotype in NSCLC	Pancreatic cancer, NSCLC (non-small cell lung cancer)
IL-7	Inhibits cancer progression, enhances therapy	Inhibits colon cancer progression; associated with overall survival (OS) and pathological stage; boosts tumor-infiltrating lymphocytes	Improved survival with chemotherapy in colorectal cancer; increased T-cell numbers and function in lymphomas	Colon cancer, colorectal cancer, lymphomas
IL-9	Complex role in tumor immunity	Promotes both tumor growth and antitumor immunity; induces apoptosis and enhances CTL activity in solid tumors; promotes metastasis in lung cancer	Inhibits tumor growth in gastric, colon, and breast cancers; associated with poor prognosis in hematologic malignancies	Gastric cancer, colon cancer, breast cancer, lung cancer, hematologic malignancies
IL-15	Cancer immunotherapy	Enhances antitumor responses; IL-15 agonists inhibit tumor growth and metastasis	Shows promise in cancer immunotherapy; ongoing clinical trials	Various cancers
IL-21	Enhances antitumor responses	Enhances memory T-cell differentiation; PD-1Ab21 (IL-21 attached to anti-PD-1 antibody) improves memory stem T cells and tumor-specific CD8+ T cells	Enhanced antitumor effects; improved immune checkpoint therapy	Various cancers

**Table 2:** Summary of preclinical studies identifying the role of immunotherapy with IL-2 cytokine family in melanoma models.

Therapy/study	Mechanism	Model	Results	Reference
Nemvaleukin and anti-PD-1	Engineered IL-2–based cytokine enhancing T-cell infiltration and expansion	Humanized mice with patient-derived tumor cells and immune cells	Increased CD4+ and CD8+ T cells, slowed tumor growth, specific antitumor responses	[101]
TBI and IL-2 in ACT	Combination aimed at improving persistence and function of transferred T cells	Bearing Rag1 knock-out mice	Increased T-cell numbers but reduced activation, decreased dendritic cells	[102]
IL-2 and IL-15 in LNs	Enhancing expression of activating and inhibitory receptors on lymphocyte subsets	T, NKT-like, and NK cell lymphocyte subsets from regional lymph nodes of melanoma patients	Boosted NK-cell cytotoxicity, enhanced overall immune response	[103]
Gene electrotransfer of IL-2 and IL-12	Introducing therapeutic genes via electroporation	Murine B16.F10 melanoma model	Slowed tumor growth, complete regression in 71% of cases, increased dendritic cells and macrophages	[104]
Celastrol (CEL)	IL-2/CD25 binding inhibitor impacting T-cell activity	BALB/c nude mice	Reduced melanoma growth, increased CD8+ T cells, ineffective in T cell-deficient mice, synergistic with TNFR2 antagonist	[105]
LOAd732 oncolytic adenovirus	Engineered virus carrying immunostimulatory transgenes (CD40L, 4-1BBL, IL-2)	Human melanoma cells Mel526, Mel624 cells, melanoma-induced BALB/c nude mice	Activated dendritic cells, maintained oncolytic activity, stimulated T cells in presence of immunosuppressive cytokines	[106]
IL-4 and preadipocytes	Interaction with preadipocytes enhancing melanoma progression via exosomal interactions	B16 melanoma cells and 3T3L1 preadipocytes	Increased cell growth and metastasis-related proteins, reduced by exosome inhibitor	[107]
IL-9-engineered chimeric receptors	Fusion of IL-2 receptor extracellular domain with IL-9 receptor intracellular domain mimicking IL-9 signaling	C57BL/6J mice	Superior antitumor activity, improved T-cell function without prior lymphodepletion	[108]
SON-1210 fusion protein	Combination of IL-15 and IL-12 with a human albumin-binding domain improving pharmacokinetics and targeting	B16F10 melanoma mouse models	Reduced tumor growth, elevated IFNγ levels, increased Th1 and CTL cell counts, shifted macrophages to M1 phenotype	[109]
IL-15/IL-15Rα gene therapy	Direct tumor injection expanding NK and CD8+ T cells, combined with PD-L1-specific antibody enhancing NK-cell activation	HLA-A2 positive cell line 624-MEL	Boosted tumor cell destruction, improved PD-L1 blockade therapy effectiveness	[110]
Intranasal IL-15/IL-15Rα N. caninum	Modified N. caninum producing IL-15/IL-15Rα reducing lung metastases	B16F10 lung metastasis mouse model	Decreased metastasis coverage, boosted immune cell levels, shifted macrophages to M1 phenotype	[111]

**Table 3:** Summary of clinical studies indicating the utilization of IL-2 cytokine family as a treatment for melanoma.

Reference	Type of study	Trial number	Patients	Intervention	Type of targeted IL-2 family receptor	Comparison to conventional therapies	Adverse events	Outcome
[128]	Phase II clinical trial	NCT02581930	Patients with metastatic melanoma	Ibrutinib, 840 mg PO QD	IL-2 inducible kinase	Compared to conventional therapies, PD-1 and MAPK inhibitors	Fatigue, hyponatremia, anorexia, nausea, vomiting, diarrhea, constipation, hypoalbuminemia, dysgeusia	No antitumor responses; PFS of 1.3 months. Treatment showed no significant clinical benefit
[129]	Phase II clinical trial	NCT01416831	Patients with metastatic melanoma	SBRT + IL-2 600,000 IU/kg TDS	IL-2	Compared to IL-2 monotherapy	Hypotension requiring vasopressor support, pulmonary capillary leak with hypoxemia, fever, rigors, myalgias, arthralgias, pruritus, erythematous rash, diarrhea, nausea, electrolyte abnormalities, elevations of hepatocellular enzymes, azotemia, peripheral neuropathy, mental status changes, and immune-mediated hypothyroidism	ORR: 54% (SBRT + IL-2) vs. 35% (IL-2 monotherapy); DCR: 75% vs. 60% (no significant difference in progression-free or overall survival)
[136]	Phase III clinical trial	NCT00200577	Stage III melanoma patients	TIL (3 ml/min) + IL-2 (150 U/ml)	IL-2	Compared to abstention	Blood and lymphatic system disorders, cardiac disorders, ear and labyrinth disorders, endocrine disorders, gastrointestinal disorders, musculoskeletal and connective tissue disorders, renal disorders, psychiatric disorders, nervous system disorders, reproductive disorders	No significant difference in DFS (57.7% in TIL + IL2 and 43.5% abstention) or OS (65.4% in TIL + IL2 and 52.2% abstention)
[137]	Retrospective cohort study	—	Confirmed stage IV or unresectable stage III melanoma	Systemic PD-1 inhibitor + intralesional treatment IL-2 (PROLEUKIN) 3–9 MIU	IL-2	Compared to PD-1 inhibitor monotherapy	Chills, aching limbs, and fever, peripheral polyneuropathy with dysesthesia	3 complete responses, 3 partial responses, 3 progressive diseases; Therapy was well tolerated
[133]	Retrospective cohort study	—	Confirmed stage IV or unresectable stage III melanoma patients	Systemic PD-1 inhibitor + intralesional treatment IL-2 (PROLEUKIN) 3–18 MIU	IL-2	Compared to PD-1 inhibitor monotherapy	—	74% response in locoregional metastases; 37% response in distant organ metastases; Improved PFS and OS associated with absence of active distant metastases, locoregional response, increased AEC, and CD8+ TIL influx; therapy was well-tolerated
[130]	Retrospective cohort study	—	Metastatic melanoma patients	High-dose IL-2 (600,000–720,000 U/kg/dose)	IL-2	Compared to no subsequent systemic therapy	Hypothyroidism, arthralgia/arthritis, neuropathy, vitiligo, premature ventricular contractions, normal pressure hydrocephalus 4o	Long-term PFS of 5+ to 30+ years; prolonged PFS as the only systemic therapy
[132]	Retrospective cohort study	—	In-transit melanoma	Intralesional IL-2 therapy (5 MIU/ml)	IL-2	Compared to no intralesional therapy	Localized inflammation, flu-like symptoms, fever, myalgias, bacterial pneumonia	44.6% complete response rate; 65.5% had durable response; median time to recurrence: 10.5 months; recurrence often associated with distant metastases
[131]	Phase III clinical trial	NCT01884961	Metastatic melanoma	High-dose interleukin-2 + radiotherapy	High-dose IL-2 (not a specific receptor, but uses IL-2)	—	Hypothyroidism, arthralgias/arthritis, vitiligo, neuropathy, premature ventricular contractions, normal pressure hydrocephalus	Partial response in 15.7% of patients, stable disease in 36.8%, disease control rate of 52.6% after median follow-up of 39.2 months. Immunological responses in 84.2% of patients. Increased levels of IL-8 and IL-10 and circulating effector memory CD4+ and intratumoral CD8+ T cells observed. Overall well-tolerated with grade 1–2 toxicities
[134]	Phase Ib clinical trial	NCT02748564	Metastatic melanoma	Pembrolizumab: 200 mg IV every 3 weeks. High-dose IL-2: 6000 IU/Kg, 60,000 IU/Kg, or 600,000 IU/Kg IV bolus TDS	High-Dose IL-2	Compared to standard treatments	Nausea, rash, adrenal insufficiency, dyspnea	MTD not reached; 1 partial response in 9 patients (11%)
[135]	Phase I/Ib	NCT02452268	Melanoma patients	NIZ985 1 µg/kg (recombinant heterodimer comprising physiologically active IL-15 and IL-15 receptor α) as monotherapy and combined with spartalizumab (anti-PD-1 mAb)	IL-15 receptor α	—	Injection site reaction, fatigue, nausea, decreased appetite, chills, vomiting, abdominal pain, arthralgia, pyrexia, dizziness, influenza-like illness, diarrhea, myalgia, headache, dehydration, anemia, back pain, constipation, edema peripheral, weight decreased, dyspnea, hypomagnesemia, abdominal distension, hyponatremia, pruritus, pain in extremity, lymphocyte count decreased	Antitumor activity observed, with disease control rates of 45% in anti-PD-1-sensitive and 20% in anti-PD-1-resistant tumor type cohorts; best overall responses were stable disease (monotherapy) and partial response (combination therapy)

*Note:* PFS, median progression-free survival.

Abbreviations: DFS, disease-free survival; ORR, objective response rate; OS, overall survival; SBRT, stereotactic body radiation therapy; TIL, tumor-infiltrating lymphocytes.

## Data Availability

Data sharing is not applicable to this article as no datasets were generated or analyzed during the current study.

## Nomenclature

TRM cells: Tissue-resident memory T cells
SC: Skin cancer
IL-2: Interleukin-2
TME: Tumor microenvironment
CM: Conditioned media
NETs: Neutrophil extracellular traps
EVs: Extracellular vehicles
AnxA1: Neutrophil-derived annexin A1
TLSs: Tertiary lymphoid structures
ICI: Immune checkpoint inhibitors
TCR: T cell receptor
MAA: Melanoma-associated antigen
DCs: Dendritic cells
TAMs: Tumor-associated macrophages
T-regs: T-regulatory cells
MDSCs: Myeloid-derived suppressor cells
MMPs: Matrix metalloproteinases
EMT: Epithelial–mesenchymal transition
IL-2R: IL-2 receptor
Th2: T-helper 2
Tfh: T follicular helper
NK: Natural killer
sIL-2R: Soluble IL-2 receptor
NSCLC: Non-small cell lung cancer
TSCM: Memory stem T cells
DCD: Dermcidin
rhil-2: Recombinant human IL-2
TBI: Total body irradiation
LNs: Lymph nodes
CEL: Celastrol
PFS: Progression-free survival.
